# Environmental and systems‐based safety in the obstetric operating room: A narrative review

**DOI:** 10.1002/pmf2.70488

**Published:** 2026-10-21

**Authors:** Zoya Amer, Sanjana Kantipudi, Lauren Hennis, Megan D. Whitham

**Affiliations:** ^1^ Virginia Tech Carilion School of Medicine Roanoke Virginia USA; ^2^ Department of Obstetrics and Gynecology Carilion Clinic Roanoke Virginia USA; ^3^ Department of Obstetrics and Gynecology Virginia Tech Carilion School of Medicine Roanoke Virginia USA

**Keywords:** cesarean delivery, ergonomics, human factors, obstetric emergencies, operating room safety, patient safety, teamwork

## Abstract

Cesarean delivery requires an operating room (OR) that supports maternal surgery and anesthesia while preparing for fetal‐to‐newborn transition and, when needed, concurrent maternal and neonatal resuscitation. This narrative review applies a human factors framework to noise, information displays, medication and equipment organization, layout, communication, ergonomics, and emergency preparedness. These factors are examined in relation to hearing the awake patient's concerns, recognizing maternal or fetal deterioration, accessing hemorrhage treatment, and coordinating newborn assessment without interrupting maternal care. Standardized layouts, structured communication, cognitive aids, and simulation may improve care processes, but evidence for their effects on maternal and neonatal morbidity varies, and some findings are extrapolated from non‐obstetric surgery. Environmental safety initiatives should therefore assess both the reliability of cesarean care and patient outcomes, including maternal morbidity, newborn thermal status, and the maternal birth experience.

## 1 INTRODUCTION

Cesarean delivery is among the most common major surgical procedures performed in the United States and remains a central focus of obstetric quality and safety initiatives [1, 2]. Although improvement efforts have emphasized indications, technique, anesthesia management, and perioperative protocols, less attention has been directed toward the environment in which obstetric surgery occurs. The obstetric operating room (OR) must accommodate maternal surgery and anesthesia, fetal assessment before birth, and newborn assessment after delivery. At birth, maternal surgery continues while a separate neonatal team assumes responsibility for the newborn; deterioration in either patient may require escalation without interrupting care of the other [3, 4, 5, 6].

During urgent cesarean delivery, noise, monitor visibility, supply access, and role clarity determine how readily teams can share fetal concerns, follow maternal hemodynamics, prepare hemorrhage treatment, and coordinate delivery. The same room must then accommodate newborn care alongside uterine repair and continued maternal monitoring [3, 4, 5, 6, 7, 8]. Although these environmental challenges occur in other operations, their obstetric significance lies in coordinating this transition between one operative patient and two simultaneous care pathways.

This narrative review examines how environmental and ergonomic features support or impede cesarean care, distinguishing maternal and neonatal outcomes from team and workflow measures. Table 1 summarizes the evidence; Table 2 translates the narrative into practical actions and intended maternal or neonatal safety aims for labor and delivery teams.

**TABLE 1:** Summary of evidence on environmental and systems factors affecting obstetric operating room safety.

Domain	Key evidence	Main takeaway	References
Obstetric OR environment	Few studies specifically examine the physical OR environment during cesarean delivery.	Obstetric OR environmental safety is important but understudied.	[9]
Noise in obstetric OR	Cesarean delivery noise frequently exceeds recommended levels. High noise burden may reflect baseline ambient noise rather than only urgency or shift timing.	Noise is a modifiable communication and cognitive‐load hazard.	[4, 7]
Noise in general OR/delivery settings	Broader OR and delivery‐room literature links noise with impaired performance, stress, and possible maternal‐fetal effects.	Quiet periods during critical phases may improve communication.	[3, 10, 11]
Temperature	Higher OR temperature reduced neonatal hypothermia in cesarean studies, but effects on broader maternal/neonatal outcomes were mixed.	Thermal management affects neonatal safety, but optimal targets remain uncertain.	[12, 13]
Door openings/traffic	Door openings increase airborne bacterial load and may be associated with higher SSI risk.	Traffic control may reduce contamination and distraction.	[14, 15]
Distractions/interruptions	OR disruptions and case‐irrelevant conversations are associated with errors, stress, workload, impaired teamwork, and longer operative time.	Reducing interruptions may improve team performance and safety.	[8, 16, 17]
Occupational hazards	Surgical smoke, anesthetic gases, radiation, and other exposures may affect OR personnel, including reproductive health.	Environmental safety includes clinician health and sustainability of the workforce.	[18, 19, 20]
Ergonomics	Surgeons commonly experience musculoskeletal pain from posture, repetitive tasks, and instrument design.	Ergonomic design may reduce fatigue, injury, and workflow inefficiency.	[21, 22]
Safety checklists/bundles	Surgical checklists, cesarean‐specific checklist guidance, hemorrhage bundles, and maternal early warning tools support readiness and escalation.	Structured tools can standardize communication and emergency response.	[5, 23, 24, 25]
Simulation/CRM training	Simulation and CRM training can improve knowledge, communication, safety climate, and identification of workflow barriers.	Team training should test systems, roles, and environment, not just individual knowledge.	[6, 26, 27, 28, 29, 30]
Team identification	Labeled surgical caps improved name recognition, teamwork, role clarity, and connection among OR team members.	Visible role/name identification may support communication.	[31, 32]

*Note*: Data are presented as narrative summaries of the cited literature.

Abbreviations: CRM, crew resource management; OR, operating room; SSI, surgical site infection.

**TABLE 2:** Practical environmental safety summary for cesarean delivery.

Domain	Action in the obstetric OR	Maternal or neonatal safety aim
Noise and patient communication	Use quiet periods during induction of general anesthesia, delivery, hemorrhage escalation, and newborn resuscitation; preserve essential alarms and the patient's voice.	Hear maternal symptoms and urgent maternal, fetal, or newborn updates [3, 4, 6, 8, 33, 34].
Support person and room traffic	Agree on support‐person location and device use; keep maternal access and the newborn handoff route clear.	Support the birth experience while limiting distraction and crowding [35].
Maternal and fetal information	Keep maternal monitoring visible to anesthesia; communicate actionable fetal changes before birth and newborn status after delivery.	Maintain maternal surveillance through the transition to two care teams [6, 25].
Medications and infusions	Use predictable storage, labeled syringes, visible allergies, and explicit infusion identification; read back urgent orders.	Reduce medication confusion during hemorrhage, hypertensive emergencies, and anesthesia care [5, 24].
Layout and emergency access	Protect routes to the maternal airway, sterile field, hemorrhage cart, blood products, and neonatal warmer.	Enable concurrent maternal stabilization and newborn resuscitation [5, 6, 26].
Positioning and ergonomics	Plan transfer assistance, protect neuraxial and vascular lines, and adjust table and instrument positions before incision when feasible.	Support safe maternal transfer and sustained exposure for delivery and uterine repair [21, 22].
Briefing and role identification	Name maternal and neonatal leads; review urgency, hemorrhage risk, anesthesia plan, allergies, neonatal needs, and contingencies.	Clarify responsibility for both patients, including when additional responders arrive [5, 6, 25, 31, 32].
Newborn handoff and warmth	Confirm neonatal attendance and equipment readiness; agree on the handoff and cord‐clamping plan and prepare thermal care.	Support prompt newborn assessment and prevention of hypothermia [6, 12, 13, 25, 26].
Emergency escalation	Use visible maternal early‐warning and hemorrhage pathways; assign arriving responders and protect maternal and neonatal work zones.	Support recognition and response during maternal deterioration or simultaneous emergencies [5, 24].
Simulation and debriefing	Rehearse urgent cesarean delivery with maternal hemorrhage and newborn resuscitation; identify supply, access, and communication failures.	Test whether the local setup supports both patients under time pressure [6, 26, 27, 28, 29, 30].
Local capability and transfer	Identify anticipated needs for blood products, specialist surgery, and neonatal care; arrange transfer when local capability is insufficient.	Match high‐risk maternal and neonatal needs to an appropriate facility [36].

*Note*: Safety aims describe the rationale for the actions; they are not claims that each action independently reduces maternal or neonatal morbidity. Bracketed numbers refer to the manuscript references. Adapt this summary to local cesarean, hemorrhage, anesthesia, and neonatal protocols; it is not a validated checklist.

Abbreviation: OR, operating room.

## 2 PHYSICAL ENVIRONMENT AND SYSTEMS DESIGN

Physical design for cesarean delivery must preserve access to the maternal airway and operative field while supporting fetal assessment before birth and newborn handoff afterward. A layout that facilitates one task but obstructs another may delay coordinated treatment when maternal hemorrhage and newborn resuscitation occur together.

In the cesarean OR, maternal and fetal updates, the awake patient's voice, and communication with the neonatal team compete with alarms, suction, room traffic, music, fans, and nonessential conversation [3, 4, 8]. Excessive noise can impair communication clarity, increase cognitive workload, and disrupt closed‐loop communication during critical phases such as induction of general anesthesia or incision, delivery, hemorrhage management, and neonatal resuscitation [3, 4, 6, 8, 26]. In a prospective analysis of 312 cesarean deliveries, more than 60% of case time exceeded the 60‐dB threshold considered safe, indicating that ambient background noise, rather than case acuity alone, drives exposure. A behavioral program combining staff education with audiovisual (traffic‐light) feedback reduced intraoperative noise by roughly 3.6 dB(A) over the whole procedure and was associated with lower patient‐reported stress [4, 33].

Quiet periods should be tied to obstetric tasks: induction of general anesthesia, delivery and newborn handoff, and escalation of maternal hemorrhage or neonatal resuscitation. Limiting nonessential conversation, music, and room entry during these periods should preserve essential audible alarms, clinical read‐backs, and communication with the patient. Family presence during cesarean delivery may provide emotional support; however, additional support persons and unrestricted use of personal electronic devices may increase noise, crowding, distraction, and privacy concerns. Units should establish clear expectations regarding the number and location of support persons, photography, video recording, video calls, and circumstances in which family presence or device use must be restricted, particularly during induction of general anesthesia, maternal deterioration, neonatal resuscitation, or other critical phases of care [35]. For the awake patient, this approach should support a birth environment in which symptoms and concerns can be heard alongside urgent clinical updates.

Before delivery, teams integrate maternal vital signs, fetal heart rate information, anesthesia management, surgical progress, and medication administration. After birth, newborn assessment adds a separate stream of information while maternal surgery and monitoring continue [3, 6, 23]. When displayed across separate monitors or role‐specific workspaces, situational awareness may become fragmented. This is especially relevant during urgent cesarean delivery or hemorrhage, when delayed recognition of maternal instability, fetal compromise, or active interventions may impair team response [3, 8, 23].

Visual information systems should support this change in obstetric information needs without diverting attention from ongoing maternal care. Anesthesia should retain primary access to detailed maternal physiologic monitoring—including airway status, ventilation parameters, end‐tidal carbon dioxide, anesthetic medications, and infusion data—while shared displays should present only information relevant to obstetric and nursing teams, such as maternal blood pressure trends, oxygenation, estimated blood loss, transfusion status, and key procedural milestones. Duplicate displays may facilitate co‐visualization across workspaces without disrupting discipline‐specific workflows [6, 26]. Before birth, clinicians responsible for fetal monitoring should communicate changes that alter delivery urgency or neonatal preparation. At birth, shared updates should shift to newborn status while continuing to report maternal blood loss, oxygenation, and hemodynamics.

Visual tools such as OR whiteboards, role boards, or shared displays can identify team members, document allergies, track active infusions, note hemorrhage risk, and display emergency plans [3, 6, 23]. For cesarean teams, a visible hemorrhage plan and infusion list can support continuity as attention shifts to delivery and newborn care, reducing reliance on repeated verbal clarification.

Cesarean delivery may require rapid treatment of maternal hemorrhage or hypertension alongside anesthetic management, creating competing medication tasks under time pressure [3, 5, 24]. Patients may receive antibiotics, uterotonics, antihypertensives, anesthetic agents, vasopressors, magnesium sulfate, and blood products in rapid sequence. Risk increases when storage is inconsistent, labeling is unclear, allergies are not visible, or active infusions are not obvious.

Medication safety infrastructure should include consistent locations for storage of medications such as uterotonics, antihypertensives, vasopressors, antibiotics, magnesium sulfate, and emergency medications; clearly labeled syringes; visible allergy information; and explicit identification of active infusions [5, 24]. During cesarean hemorrhage or a hypertensive emergency, these measures should help the team distinguish the intended drug and active infusion while anesthesia and surgery proceed in parallel.

During cesarean delivery, table height, patient body habitus, assistant positioning, and instrument placement affect exposure for delivery, uterine repair, and hemorrhage control. Ergonomic planning must also preserve anesthesia access to the patient and an unobstructed newborn handoff route. Prolonged static posture, repetitive motion, awkward positioning, and physically demanding retraction may contribute to cumulative musculoskeletal risk among surgeons and proceduralists, among whom pooled 12‐month pain prevalence reaches roughly 60% for the neck, 52% for the shoulder, and 49% for the back, and 12% report needing a leave of absence, practice modification, or early retirement [21, 22]. These estimates describe surgeons and proceduralists broadly. They support attention to staff strain but do not establish that ergonomic changes during cesarean delivery reduce maternal or neonatal morbidity.

Surgical setup should be tailored to anticipated case demands. Instrument trays and back‐table organization should prioritize predictable placement of high‐use instruments, separation of routine and emergency supplies, and rapid access to items needed for delivery, uterine repair, hemorrhage control, and neonatal handoff [5, 6, 24, 26]. OR table height and patient positioning should be adjusted before incision when feasible to reduce surgeon and assistant strain, particularly for cases involving elevated body mass index (BMI), difficult exposure, or anticipated prolonged retraction [21, 22].

Cesarean OR layout should allow the newborn to reach the neonatal team without blocking maternal airway access, hemorrhage treatment, or blood‐product delivery. Movement between the sterile field, anesthesia workspace, warmer, medication area, and exit should be planned for simultaneous maternal and neonatal care. Hemorrhage carts, emergency airway equipment, neonatal resuscitation supplies, and point‐of‐care testing supplies should be placed in predictable and immediately accessible locations [5, 6, 26].

Every obstetric OR case should include anticipatory room planning, although the degree of preparation should reflect the expected clinical complexity. Standardized equipment placement, confirmation of essential supplies, assignment of team roles, and verification of emergency equipment readiness should occur routinely. For patients with suspected placenta accreta spectrum, anticipated hemorrhage, or expected neonatal resuscitation, additional preparation may include pre‐positioning hemorrhage supplies, confirming blood product availability, ensuring neonatal equipment readiness, arranging interventional radiology support when appropriate, and clarifying contingency plans before incision [5, 6, 26]. Planning should therefore identify where additional maternal and neonatal responders will work and how blood products, airway equipment, and the newborn will move through the room if the case escalates.

## 3 TEAM COMMUNICATION AND COORDINATION IN ROLE‐SPECIFIC ENVIRONMENTAL INTERACTIONS

Cesarean teamwork requires an explicit plan for the transition at birth. Obstetric and anesthesia clinicians continue surgery and maternal physiologic surveillance, nursing staff coordinates supplies and information, and neonatal clinicians assume newborn assessment. The briefing should identify who communicates maternal status, who receives newborn updates, and how urgent concerns reach the relevant team. This division of responsibility should remain visible when additional responders enter, so that attention to the newborn does not interrupt maternal surveillance. Cesarean‐specific checklists address coordination of care for both patients [25].

Communication failures and disruptions in surgical flow have been associated with increased workload, impaired teamwork, and error risk in operative environments, and in a meta‐analysis such disruptions accounted for approximately 22% of total operative time [6, 8, 16, 17, 23]. These general operative findings provide a rationale for reducing interruptions but do not quantify cesarean‐specific risk. In obstetrics, the relevant tasks include communicating changes in delivery urgency, maternal instability, and newborn readiness across separate teams.

Closed‐loop communication should be used for critical information, including medication orders and dosing, blood product requests, maternal status changes, neonatal concerns, and emergency protocol activation. Read‐back protocols should be used for high‐risk medications and time‐sensitive verbal orders, particularly during hemorrhage, hypertensive emergencies, induction of general anesthesia, magnesium administration, and massive transfusion. Role identification at the start of each case can reduce confusion, particularly when team members are unfamiliar with one another or additional personnel enter during urgent cesarean delivery or hemorrhage [6, 16, 23]. Briefings should be concise but include the indication for cesarean delivery, urgency level, hemorrhage risk, anesthesia plan, neonatal needs, allergies, active infusions, and role assignments. Debriefings should identify equipment, layout, communication, staffing, or medication barriers that affected care [5, 6, 23, 26].

During cesarean delivery, the anesthesia team maintains maternal physiologic stability, manages neuraxial or general anesthesia, administers medications, and communicates hemodynamic changes. Under neuraxial anesthesia, this role also requires listening to the awake patient and evaluating reports of pain or discomfort. Noise, poor monitor visibility, and separation from the sterile field can hinder both maternal status updates and communication between the patient, anesthesia clinician, and surgeon [8, 24, 34].

To support shared situational awareness, maternal status updates should be incorporated into the surgical workflow through predefined verbal triggers, such as significant hypotension, escalating vasopressor requirement, oxygen desaturation, quantitative blood loss thresholds, massive transfusion activation, or concern for airway instability [5, 8, 24]. These triggers keep maternal deterioration visible to the surgical team even when delivery and newborn assessment dominate room activity.

The circulating nurse often functions as the central communication and coordination hub, linking the sterile field, anesthesia team, neonatal team, blood bank, supply areas, and documentation systems [5, 16]. During cesarean delivery, competing requests for maternal medications, blood products, surgical supplies, and neonatal equipment can overload this coordinating role. Patient transfer, positioning, and preparation also carry safety risks for both patients and staff, particularly with elevated BMI, limited mobility, neuraxial anesthesia, or urgent operative delivery [3, 4, 21, 22].

Patient transfer protocol communication should specify required personnel, transfer devices, protection of neuraxial and vascular lines, positioning responsibilities, and adaptations for elevated BMI or limited mobility [3, 4, 21, 22]. During induction of general anesthesia, incision, delivery, hemorrhage management, and neonatal resuscitation, nonessential conversation, supply requests, and room entry should be limited so nursing staff can prioritize essential safety tasks. Post‐event debriefings should specifically ask nursing staff whether supply access, documentation demands, room traffic, unclear requests, or staffing limitations affected care, because these workflow barriers may not be visible to all team members during the event.

At delivery, the focus of care expands from maternal surgery to simultaneous maternal and neonatal management. Neonatal clinicians may enter the OR, receive a handoff, prepare resuscitation equipment, assess the newborn, and coordinate transfer while the obstetric and anesthesia teams continue maternal care [6, 26]. Limited space, variable neonatal team positioning, unclear role assignments, distance from the warmer, and equipment readiness may affect response time, especially in preterm delivery, fetal distress, congenital anomalies, or neonatal resuscitation [6, 26].

Neonatal activation criteria and role assignments should be defined before delivery whenever possible. The neonatal warmer should be positioned close enough for rapid handoff and communication of neonatal status, but far enough to preserve sterility, maternal access, and movement for anesthesia and nursing staff [6, 26]. Communication pathways should specify who announces delivery timing, whether and how delayed cord clamping will occur, who provides prenatal or intrapartum history, and how neonatal status is communicated back to the maternal team.

## 4 ENVIRONMENTAL SAFETY IN OBSTETRIC EMERGENCIES

During an urgent cesarean delivery, the room may need to support delivery for fetal compromise, maternal stabilization, and newborn resuscitation in rapid succession or at the same time. The challenge is to preserve the maternal airway and operative field while accommodating neonatal care and newly arriving responders. Emergency preparedness should therefore be tested against concurrent maternal and neonatal needs, rather than assuming that a setup adequate for a routine case will remain functional during escalation.

Preparedness should therefore be treated as an environmental and systems‐based intervention. Obstetric hemorrhage bundles and maternal early warning tools translate preparedness into measurable benefit. Implementation of a Maternal Early Warning Trigger tool significantly reduced Center for Disease Control and Prevention (CDC)‐defined severe maternal morbidity and composite maternal morbidity, with no corresponding change at nonpilot control sites, and, in general surgical populations, surgical safety checklists have been associated with reductions in postoperative death (1.5% to 0.8%) and inpatient complications (11.0% to 7.0%) [5, 23, 24, 25]. What distinguishes the emergency from the routine case is not the need for these resources but the conditions under which they must be accessed, such as additional personnel unfamiliar with the room, crowding, obstructed movement paths, competing work zones, and the need for rapid equipment retrieval under time pressure.

Communication structure is equally important during crisis conditions. Emergencies often create competing conversations among teams, including requests for instruments, medication administration, blood product activation, maternal status updates, and neonatal preparation. Noise, crowding, and overlapping tasks may increase the risk that essential information is missed or directed to the wrong person. Closed‐loop communication should therefore be used for high‐risk medication orders, blood product requests or massive transfusion activation, maternal status changes, and neonatal concerns [6, 8, 16]. Clear leadership and standardized triggers, such as quantitative blood loss and vital sign thresholds, may reduce ambiguity and align the team's understanding of urgency [5, 6, 24].

Physical layout and equipment access can either support or delay emergency response. Inconsistent supply locations, cluttered workspaces, and obstructed pathways may delay access to uterotonics, airway equipment, hemorrhage carts, neonatal resuscitation supplies, instruments, or blood products [8, 16]. Standardized equipment placement across obstetric operating rooms may reduce reliance on memory and room‐specific workarounds, particularly when additional personnel enter during emergencies [5, 8, 16]. Clear movement pathways between the sterile field, anesthesia workspace, medication area, neonatal warmer, and room exit are especially important during hemorrhage, conversion to general anesthesia, crash cesarean delivery, or neonatal resuscitation.

Cognitive aids and simulation‐based training can help translate emergency preparedness into routine practice. Hemorrhage algorithms, maternal early warning pathways, role‐based checklists, and brief emergency huddles may reduce cognitive burden by making key steps visible during high‐acuity events [5, 23, 24]. Simulation allows teams to test whether protocols, equipment access, room setup, communication pathways, and role assignments function reliably under realistic conditions [6, 26]. Multi‐professional simulation‐based obstetric team training probably reduces cesarean deliveries (291 vs. 368 per 1000 women) and neonatal trauma from shoulder dystocia, though effects on maternal and perinatal mortality remain uncertain [27]. These findings concern obstetric emergency training broadly and do not isolate the effect of cesarean OR design. For cesarean teams, simulation and debriefing should test simultaneous maternal hemorrhage and newborn resuscitation, identify which access or communication failures occurred, and guide changes to the local setup.

## 5 DISCUSSION, LIMITATIONS, AND FUTURE DIRECTIONS

The obstetric significance of environmental design is its ability to support maternal surgery and newborn transition within the same room. Noise control, visible information, organized supplies, and clear responsibilities address different parts of that coordination, including hearing maternal symptoms, recognizing deterioration, and making hemorrhage or neonatal equipment accessible [3, 4, 5, 6, 7, 8, 16, 21, 22]. The expected safety benefit is a more reliable response to maternal and neonatal needs; it should not be equated with proven reductions in morbidity for every intervention. Staff workload and workflow measures remain useful for identifying barriers, but patient outcomes are needed to establish clinical benefit.

Environmental safety and protocolized care are complementary, rather than distinct. Obstetric hemorrhage bundles, maternal early‐warning pathways, and cesarean‐specific surgical safety checklists structure recognition, escalation, and coordination. Their implementation requires an environment that supports the relevant obstetric tasks: predictable supply locations, visible cognitive aids, and consistent room setup [5, 24, 25]. Conversely, environmental interventions are most durable when embedded in protocol. Framing OR design as the substrate that makes protocols executable clarifies that standardized workflows and standardized environments are two halves of the same high‐reliability strategy.

The current literature remains limited. Much of the evidence is observational, simulation‐based, or extrapolated from non‐obstetric surgical settings, and there is limited obstetric‐specific research on OR layout, visual information systems, cognitive aids, ergonomics, and active noise reduction programs. This gap between process and outcome is illustrated by ambient OR temperature. Two obstetric randomized trials at the same institution showed that raising OR temperature reduces neonatal hypothermia—from 50% to 35% at 23°C and from 43% to 18% at 24°C—yet neither detected a reduction in neonatal morbidity, demonstrating that an environmental change can reliably improve neonatal thermal status without a proven improvement in hard clinical outcomes [12, 13]. Future cesarean studies should pair clinical outcomes, such as severe maternal morbidity, transfusion requirements, neonatal hypothermia, resuscitation needs, and maternal pain or birth experience, with the care processes each environmental intervention is intended to improve. Relevant process measures include time to hemorrhage activation and blood‐product administration, equipment retrieval delays, medication errors, and communication failures. Reporting both levels would help distinguish a more efficient workflow from demonstrated maternal or neonatal benefit; staff injury and workload should be evaluated separately.

These principles are not confined to well‐resourced centers. Many of the highest‐yield interventions (quiet periods during critical phases, consistent medication and hemorrhage‐supply organization, standardized room setup, structured briefings/debriefings, and visible cognitive aids) are low‐cost and require behavioral and organizational change, rather than capital investment. Tools validated in low‐resource settings, including the WHO Surgical Safety Checklist (associated with reduced mortality [RR ∼0.77] and complications [RR ∼0.56] in low‐ and middle‐income countries) and low‐cost early‐warning devices such as the CRADLE Vital Sign Alert, show that structured environmental and systems interventions are feasible where they may matter most [37, 38]. Where local resources cannot support definitive management of anticipated high‐acuity cases, such as placenta accreta spectrum, anticipated massive hemorrhage, or extreme prematurity, environmental preparedness extends to a maternal levels‐of‐care framework: early risk identification, activation of transfer protocols, and antepartum transfer to a facility with the requisite blood‐bank, surgical, interventional, and neonatal capabilities before delivery [36]. In this context, the most important ‘environmental’ decision may be recognizing when the safest operating room is at another institution.

Obstetric quality improvement should select environmental changes around a defined cesarean care problem: missed maternal updates, delayed hemorrhage supplies, an obstructed newborn handoff, or difficulty hearing the awake patient. Teams can then use briefings, local simulation, and case review to test whether the change improves the relevant process and assess associated maternal or neonatal outcomes. Table 2 provides a practical summary for this local work; it does not replace clinical protocols or establish the independent effectiveness of each action.

## 6 CONCLUSION

Environmental safety in cesarean delivery depends on coordinating continuing maternal surgery and anesthesia with fetal assessment before birth and newborn care afterward. Noise, monitoring, medication organization, layout, and ergonomics should be evaluated against those obstetric tasks, including the possibility of simultaneous maternal and neonatal emergencies. Practical changes may improve care reliability, but claims of clinical benefit must distinguish direct maternal and neonatal outcome evidence from workflow improvements and findings extrapolated from other surgical settings.

## AUTHOR CONTRIBUTIONS

**Zoya Amer**: Writing—original draft; writing—review and editing; project administration. **Sanjana Kantipudi**: Writing—original draft; writing—review and editing; project administration. **Lauren Hennis**: Conceptualization; writing—review and editing; project administration; supervision. **Megan D. Whitham**: Conceptualization; writing—review and editing; project administration; supervision.

## CONFLICT OF INTEREST STATEMENT

The authors declare no conflicts of interest.

## FUNDING INFORMATION

The authors received no specific funding for this work.

## ETHICS STATEMENT

This article is a narrative review of previously published literature and does not involve human participants or animals.

## Data Availability

Data sharing is not applicable to this article because no new data were created or analyzed in this narrative review.
